# Personalized Culinary Medicine: Qualitative Analyses of Perceptions from Participants in Action and Contemplation Stages of Change Through a One-Year Bi-Center Randomized Controlled Trial

**DOI:** 10.3390/nu17040704

**Published:** 2025-02-16

**Authors:** Adi Finkelstein, Maggi A. Budd, Brianna E. Gray, Jacob Mirsky, Amir Tirosh, Rani Polak

**Affiliations:** 1 Selma Jelinek School of Nursing, Faculty of Life and Health Sciences, Jerusalem College of Technology, Jerusalem 9548307, Israel; afinkels@g.jct.ac.il; 2 Department of Spinal Cord Medicine, VA Boston Healthcare System, Boston, MA 02130, USA; margaret.budd@va.gov; 3 Department of Psychiatry, Harvard Medical School, Boston, MA 02115, USA; 4 Translational and Clinical Research Centers, Massachusetts General Hospital, Boston, MA 02114, USA; bgray4@mgh.harvard.edu; 5 Division of General Internal Medicine, Massachusetts General Hospital, Boston, MA 02114, USA; jmirsky@mgh.harvard.edu; 6 Division of Endocrinology, Diabetes and Metabolism, Sheba Medical Center, Ramat Gan 5262000, Israel; amir.tirosh@sheba.health.gov.il; 7 Sackler School of Medicine, Tel-Aviv University, Tel Aviv 6997801, Israel; 8 Department of Physical Medicine and Rehabilitation, Harvard Medical School, Spaulding Rehabilitation Hospital, Boston, MA 02130, USA; 9 Sheba Center of Lifestyle Medicine, Sheba Medical Center, Ramat Gan 5262000, Israel

**Keywords:** home cooking, stages of change, personalized medicine, lifestyle medicine, culinary medicine

## Abstract

**Background**: A high-quality diet is linked to cardiometabolic risk reduction. Culinary medicine interventions are effective in improving nutrition and health outcomes. While personalized nutrition is usually related to improving patient outcomes through knowledge about gene-nutrient interactions, tailoring interventions based on participant motivation and biopsychosocial environment may improve outcomes. The stage of change framework categorized participants based on current behaviors and intentions for future behaviors. Our goal was to assess participant perceptions regarding accomplishments, challenges, and needs up to one year following a culinary medicine program according to their stage of change at entry. **Methods**: Participant perceptions were collected at (1) the intervention end (open-ended questionnaire), (2) six months (semi-structured interview), and (3) twelve months (open-ended questionnaire). Analysis was performed inductively following a thematic analysis approach. **Results**: Twenty-four participants completed 70 perspectives (58/12 from participants who entered at a contemplation/action stage of change). Perceptions were related to (1) acquire culinary and nutritional knowledge: improve knowledge about healthy nutrition, use new recipes, and ask for hands-on cooking classes; (2) improve culinary and self-regulatory skills: improve confidence in the kitchen, expand cooking skills, organizing and planning, and creativity and pleasure; (3) adopt home cooking and healthy nutrition: adopt home-cooking habits, spreading home cooking to other family members, improve nutrition habits throughout the day, and decrease consumption of ultra-processed food; and (4) address the sustainability of health changes: achievements in maintaining long-term health changes, challenges in maintaining long-term health changes, and facilitators for a long-term change. **Conclusions**: These results provide one-year-long information about participant facilitators, barriers, and needs for making home-cooking changes categorized to the participant stage of change at program entry. This information can help reform effective personalized culinary medicine programs.

## 1. Introduction

A high-quality diet is linked to cardiometabolic risk reduction [1,2,3], resulting in the need for a global effort to educate the public about healthy eating [4,5,6]. Possessing knowledge of what constitutes a healthy diet does not always elicit lasting change [7,8]. This discrepancy between knowledge and long-term behavior change is attributed to numerous barriers, including cultural traditions surrounding food, poor planning, and underdeveloped culinary skills [9]. Culinary medicine (CM) is an emerging strategy to improve nutrition through home cooking [10]. CM interventions have been proven effective in improving nutrition and health outcomes [11]. While most CM programs are delivered in teaching kitchen facilities [12], novel programs use culinary videos [13], live remote culinary demonstrations [14] through telemedicine platforms [15,16], and also artificial intelligence support [17].

Key determinants influencing home cooking include female gender [18,19], employment, and ethnic background [19,20]. These suggest that determinants of home cooking expand beyond cooking skills and confidence [21,22,23]. Older females are generally noted to have more confidence with cooking [24] skills and more expectations from others to cook [18]. Socioeconomic status is an additional determinant affecting home cooking. Although lower-income people may cook more often at home, they do so with lower-quality food [22]. Having time and money to cook healthy can be considered a privilege more than a necessity for people with lower incomes [24] with some feeling they may not have the luxury [22]. Costs of healthy foods have been found to be a barrier for people with lower income [22]. At the same time, a motivator to prepare food at home is often to save money [25].

The short-term nutritional impact of culinary and home-cooking education is well established [26,27]. However, establishing long-term behavioral change remains a critical gap [28]. Barriers include a lack of self-motivation and self-regulation to continue healthy behavior changes [9]. Personalized nutrition is usually related to improving patient outcomes through knowledge about gene–nutrient interactions. Tailoring nutrition interventions based on an individual’s biopsychosocial factors [24], such as motivation, may promote the adoption of long-term healthy eating. High-impact, personalized nutrition and CM interventions aimed at increasing individuals’ self-efficacy, readiness to change, and ability to make dietary changes are needed. Perceptions about home cooking were widely published [13,23,29]. To better develop future CM programs, this report is the first to present participants’ perceptions throughout a one-year period following a CM program in the areas of (1) nutrition and home-cooking changes, accomplishments, and challenges, (2) effective tools and resources and long-lasting learning moments, and (3) recommendation to future CM programs

One approach to personalized nutrition intervention based on individual motivation is to utilize the stage-of-change (SOC) framework developed for smoking cessation and applied to several health behaviors, including healthy eating [30,31,32,33]. Using this framework, individuals are categorized into one of five SOCs based on their current behaviors and intentions for future behaviors, often termed “readiness for change” [34]. As applied to behaviors of home cooking, a pre-contemplator is an individual who has no intention of implementing home cooking in the foreseeable future. Individuals in a contemplation SOC think about changing home-cooking behaviors but have not implemented plans for change. Contemplators and preparers are aware of reasons for and against making changes and intend to make changes soon. Individuals in the action SOC have a plan and are currently creating a healthier pattern of behavior, but the new pattern of behavior has not been sustained for an extended period and is not yet fully integrated change into a person’s lifestyle, which would be the maintenance SOC.

The basis for intervention using the SOC involves ten processes of change (POCs) related to transitions between the stages and that are the mediators for change [34]. The POCs cross several theoretical models, apply to different SOCs, and are divided into experiential and behavioral processes. Both experiential and behavioral POCs have been predictive of progression through SOCs [30]. Experiential POCs are most effective for individuals in the contemplation SOC and include conscious raising (i.e., increased awareness of self and barriers to behavioral change), self-re-evaluation (i.e., assessing pros and cons of behaviors), and dramatic relief (i.e., experiencing and expressing emotional reactions related to the behavior change). Behavioral POCs are most effective for those in the action SOC [35], including self-liberation (i.e., selecting and committing to action and belief in one’s ability to change), counter conditioning (i.e., substituting alternatives to old behaviors), and stimulus control (i.e., observe and re-structure the environment).

While several CM programs have been described, and participants in various levels of motivation present different POCs, there are no reports about participants who were referred to different CM programs according to their motivational to adopt home-cooking habits. Our goal is improving the impact of CM programs through curricula segmented according to participants’ motivational characteristics. This report is the first to present perceptions throughout a one-year period following a CM program. We aimed to analyze one-year perceptions of participants who entered a CM program at either a contemplation or active SOC.

## 2. Materials and Methods

This is a bi-center randomized controlled trial aimed at evaluating the impact of a remote home-cooking intervention on the nutrition and weight of participants who are overweight or obese (approved by both sites’ institutional review boards, protocols #2018P002115 and 5419-18-SMC). This report follows the SRQR guidelines [36] for qualitative research.

### 2.1. Setting and Participants

Participants were recruited at Spaulding Rehabilitation Hospital (Spaulding), Boston, Massachusetts, United States, and Sheba Medical Center (Sheba), Ramat Gan, Israel. Inclusion criteria included body mass index (BMI) equal to or greater than 27.5 kg/m^2^ and equal to or lower than 35 kg/m^2^; primary food provider of the household who consumes fewer than five home-cooked lunches and dinners per week; and age 25–70 years. Participants were assigned, through simple randomization, at each site to either an intervention or control group.

Seventy-nine participants enrolled in the CM study at both sites; of them, 39 were randomized to the intervention arm. Two were recruitment failures, and three dropped out before the completion of the baseline visit. In addition, ten dropped out before visit 2 due to changes in availability around work/COVID-19 pandemic. Participant demographics are presented in Table 1 (n = 24; 12 from Spaulding and 12 from Sheba). Most were married women (68%), with a mean age of 44.17 (12.92) and above the average income (66%).

Seventy perspectives (97%) were analyzed (23 at 3 months, 24 at 6 months, and 23 at 12 months). Of them, 58 from participants who entered the study at contemplation SOC, and 12 from participants who entered at action.

### 2.2. The Culinary Coaching Telemedicine Program

The intervention group completed a 3-month culinary coaching telemedicine program. Both groups completed a 30 min nutritional counseling session at baseline and after the program. A detailed description of the culinary coaching telemedicine program has been published [15,16]. Briefly, the program includes 12 weekly, one-on-one, 30 min tele-sessions with a culinary coach. At the first session, participants identify their personal culinary and nutrition vision and 3-month goals. During each subsequent session, participants review their progress toward reaching the prior week’s culinary goals and identify goals for the coming week using brainstorming, reflection, and self-discovery processes facilitated by the provider. When participants identify a new culinary skill necessary for their progress, they are either instructed by the culinary coach through discussions or referred to specific program resources (e.g., recipe or video).

### 2.3. Data Collection

At baseline, a self-administrated questionnaire was collected, including the validated University of Rhode Island Change Assessment (URICA) [37] scale (a 32-item self-administered Likert scale questionnaire), which was modified using a factor analysis [38] to assess participants’ stages of change regarding home cooking before entering the program.

Participant perceptions after the program were collected only for the intervention group. Qualitative data were obtained between December 2019 and September 2022 at three different times: (1) at the end of the 3-month intervention (Time 2, questionnaires with open-ended questions); (2) at six months after the intervention initiation (Time 3, open semi-structured interviews through a Zoom discussion); and (3) at 12 months after the beginning of the intervention (Time 4, questionnaire with open-ended questions). Baseline data, before participants entered the program, were reported elsewhere [39]. See Supplementary Materials File S1 for questionnaire details and an interview guide. Interviews (an average of 20 min) were recorded and transcribed verbatim with removed identifying details. In the Section 3, participants are identified by their stage of change in program entry (A- action; C- contemplation) and visit number (2, 3, or 4).

### 2.4. Data Analysis

Analysis was performed inductively by the group qualitative researcher (A.F.) following reflexive thematic analysis [40] in accordance with the six-phase thematic approach guidelines [41]. Analysis began with reading and re-reading the data collected. Then, an open coding was performed inductively on data from each visit separately, and similar codes appeared. After a debriefing meeting with the principal investigator (R.P.), it was decided to continue the analysis of all the data as a single unit. We reshaped the open coding and continued to the next stage of themes produced. Analysis was followed by several discussions between A.F., R.P., and the team neuropsychologist (M.B.) until the final set of themes and sub-themes was completed. The process was exploratory, flexible, and iterative [41], consistent with reflexive thematic analysis [42]. Analysis was performed manually without the use of software or AI tools.

### 2.5. Rigor

The sample size in this qualitative study was predetermined by the quantitative arm calculation of the randomized controlled trial; however, saturation was reached (analytic generalization) [43]. Strategies to support transferability include thick description, which refers to rich, detailed information about the research setting and study participants. Study credibility was strengthened by utilizing two types of data collected (questionnaires and interviews) and rich data examples to follow the analysis, as well as multiple perspectives from researchers from different disciplines (anthropology, psychology, and medicine) who brought diverse perspectives and enriched the insights drawn from the data [44].

## 3. Results

We found four main themes: (Section 3.1) acquire culinary and nutritional knowledge (with three sub-themes); (Section 3.2) improve culinary and self-regulatory skills (with four subcategories); (Section 3.3) adopt home cooking and healthy nutrition (with four sub-themes); (Section 3.4) address the sustainability of health changes (with three sub-themes). Table 2 summarizes the themes and sub-themes with a sample quote. Additional quotes are integrated into the findings section.

### 3.1. Acquire Culinary and Nutritional Knowledge

#### 3.1.1. Improve Knowledge About Healthy Nutrition

Contemplation group participants expressed a growing understanding of what healthy food means and what food should be avoided through all three visits: “Understanding the food ingredients in each menu and ’filtering’ unhealthy products. For example, removing the skin from the chicken when roasting and covering the meat with vegetables or applying a drop of oil to prevent drying” (C-2); “I learned to choose foods based on nutrition” (C-2).

Participants from both action and contemplation specifically identified improvement in understanding food labels and food claims: “I finally learned how to prepare healthy food and check the ingredients I put in the pot. Not to put everything in the supermarket labeled ‘healthy’ or has a ‘healthy’ stigma… I am thirsty for more” (C-3); “I go to the supermarket or the market or anywhere else to buy the raw food items that feed me … Today I look at everything. I look at its ingredients if it’s a closed package” (A-3).

#### 3.1.2. Use New Recipes

Participants reported identifying and trying new recipes throughout all three visits: “I accomplished my goals…. with adding 1–2 new go-to recipes” (C-2); “[I am] cooking more often at home, trying new recipes” (C-2); “Enjoying cooking and not thinking of it as just another chore (like cleaning), trying new recipes” (C-2); “the program… helped me to cook more types of foods and use different recipes” (A-2).

One participant mentioned that using new recipes was in synergy with other program benefits, such as improved familiarity with kitchen equipment: “Familiarity with the kitchen equipment I have and a knowledge of recipes I can make at home easily” (C-4). Another participant specifically mentioned an educational process with the coach that resulted in using more recipes: “The significant part [in the program] was after I understood what a healthy food recipe is… I started by the encouragement of my coach to look for recipes myself from different websites…” (C-2).

#### 3.1.3. Ask for Hands-On Cooking Classes

A few participants shared that they could benefit from more culinary guidance, including hands-on cooking classes: “I have an interest in cooking guidance, and it was absent. There was sporadic guidance, but it wasn’t the essence of the program. For me, it was missing… knowledge, techniques, substitutions to fatty dishes, recipes, and other such things… even a hands-on cooking class” (C-3); “I want health-oriented workshops, food that is good, and techniques on how to make it” (C-3); “It wasn’t necessarily cooking class or cooking coaching; the culinary coaching was not what I expected. I thought I was getting culinary techniques and classes” (C-2); “I would add actual hands-on cooking with chef” (A-2).

### 3.2. Improve Culinary and Self-Regulatory Skills

#### 3.2.1. Improved Confidence in the Kitchen

Participants from both groups shared that they improved their confidence in the kitchen up to one year from baseline: “The success I’ve had, and the positive momentum will stay with me. The weekly goal setting and the resources I found as a result; and my confidence that I can cook healthy” (C-2); “I was very nervous about cooking before and basically cooked frozen foods if I cooked at all. Now, I cook mostly fresh foods, which makes me feel healthier. I have eaten significantly less meat also. I have been cooking fish more often” (C-2); “I learned that I have a very high ability in the kitchen”(A-2); “I think it helps with my confidence a lot, in terms of being able to cook healthier meals for myself and what to buy, like, where to find resources for recipes and things like that…” (C-3); “… cherishing what is there, looking at my strengths… this is what remains with me, something very strong” (A-3); “I found myself trying to be more accountable and creative in my diet. I cooked at home for almost every dinner and a handful of lunches. This greatly increased my confidence in the kitchen” (A-2); “It gave me the possibility to cook more, not one specific thing. Just the confidence to cook more…” (C-4); “The ability to take something I don’t know, and even if I don’t know it at all-try to dare” and “[I used to have] a lot more worries so as not to make a mistake… so today it doesn’t happen, if I fancy something or hear about something, the first thing I do is try it” (C-3).

#### 3.2.2. Expand Cooking Skills

In both groups and throughout the three visits following the program, participants talked about using diverse techniques that they had not used before, including steaming, cooking, and grilling: “I started steaming (something I was not doing before) and grilling more” (C-2); “I got a lot better at baking, and my knife skills got better. I also know better to making sauces I like” (A-3); “Roasting, baking, light frying; using a food pot–almost without oil” (C-4).

Participants from both groups also reported on new skills in cooking healthy ingredients such as vegetable legumes and other plant-based food items: “I cooked many vegetarian dishes that I could never have made before” (A-2); “Vegetables have become a very significant raw material” (A-2). In addition, participants specifically reported that the healthy ingredients substituted unhealthy food items: “When cooking, use onions, zucchini, pumpkin and other low-calorie vegetables instead of carbohydrates” (C-4); “The main and significant change is mainly in the use of legumes, lentils and less use of carbohydrates (potatoes, pasta)” (C-2); “I eat a lot more olive oil than I ever did because of the program. I substituted, I don’t eat butter or margarine anymore” (C-3).

Action group participants also reported on cooking more challenging dishes “Slow cooking with very little oil, use of cooking in the oven, combination of foods” (A-4); “I also cooked complicated dishes on weekends that I would have otherwise purchased” (A-2).

#### 3.2.3. Organizing and Planning

In both groups and throughout the three visits, participants noted that they are more organized in planning their diet: “Planning meals in advance… so that the ingredients are at home and the time needed for cooking is available. This means to plan already from the weekly shopping what I prepare that week” (C-2); “Basically, until the program, I would cook once a week, and it was always on Friday. During the week, I managed with what was left… and suddenly, I cooked according to the way I defined for myself… for some reason it worked. I just eat cooked food every day” (A-3); “Everything starts from the day of shopping, choosing the right raw materials, right combinations” (A-2).

While participants who entered the program in the action SOC generally reported being more organized and planned, participants who entered at contemplation reported using specific organizing and planning tools: “I use a meal planning tool; I’m planning all my meals for the week. That’s one thing I never did before, and I frequently use now… I do meal prep in advance” (C-3); “I definitely plan a lot more; I never did it before; it was ‘I’m hungry now.’ I use the notes on my phone and other notes apps like planning things, making grocery lists, thinking what meals I might eat” (C-3).

Participants who entered the study in contemplation also identified time-saving culinary tools that they acquired in the program, such as batch cooking and the use of leftovers: “Batch cooking was the most useful and not thinking of it as (just) making one big meal to last a few days but thinking of the ingredients … you chop and freeze… this has made me more likely to cook healthy meals” (C-2); “I started freezing leftovers instead of feeling compelled to eat them. Unfreezing is easier than over cooking” (C-2).

#### 3.2.4. Creativity and Pleasure

A few participants, mainly those who entered in the action SOC and through all three visits, noted that, following the program, they are more creative in the kitchen, including recipe substitutions: “I learned that I could make any changes I want to recipes to use all the food in my fridge, (i.e., substitute spinach for kale and chard)” (A-2); “It mainly knows how to cook, reinventing yourself in the kitchen…” (A-3); “I am expanding the repertoire to other flavors, which makes it more likely to cook at home. I am not getting bored from looking at recipes again; it’s not like learning how to fry but looking at a recipe and thinking more about it. It was very helpful” (C-3); “Taking initiative in the kitchen and thinking outside the box” (A-4); “To love to cook for health and pleasure” (A-4); “…cooking skills will stay with me as will my confidence and newly found enjoyment of cooking. I cook as a hobby now” (A-4); “I also find that one of the things that has helped me the most is just looking up recipes on New York Times cooking and improvising” (A-4). However, not all participants reported improved creativity: “My creativity was finished at some stage, and I felt I was off track again to unhealthy dishes” (A-2).

Participants mostly from the action SOC shared about enjoying cooking and the new food they eat, including other family members: “Yes, I enjoyed the new food that I cooked” (C-2); “I enjoyed the food, and so did my partner, I made new dishes mixed up our routine of the same meals and created my own dishes without recipes” (C-2); “You feel good when everyone enjoys something that you cook” (A-2); “I learned to get to know myself and the abilities I didn’t know. A good example is when I find myself coming home and happily going into the kitchen and starting to take care of myself” (A-2).

### 3.3. Adopt Home Cooking and Healthy Nutrition

#### 3.3.1. Adopt Home-Cooking Habits

Throughout the three time periods, participants from both SOCs said they cook more at home. Following the program, cooking at home became possible, easier, and more efficient and, for some, even more satisfying than before: “…I prepare most of my foods now where in the past I ordered out” (C-4); “The whole idea around cooked food is what I learned… if it’s a real meal, it’s more satisfying, gives more of a feeling that we have eaten and there is no need to eat in between… I’ve been cooking more since participating in the research” (C-3); “[Since the program]–I have been careful about foods cooked at home and trying not to buy ready-made food… and also that the food be low in salt and sugar, as well as adding physical activity” (C-4); “[Goals achieved] Cooking more regularly at home and incorporating healthier choices and portion control” (C-4); “I have been cooking at home so much! I have gotten much better at using unprocessed and raw foods. My cooking skills have improved tremendously. I am still trying to follow the Mediterranean diet and have been eating more vegetables and olive oil” (A-4).

One participant specifically shared that it becomes easier to cook with the experience s/he acquired “I cook several meals at home, and now things are easier because I’m used to it and I’m doing things faster and I have stock of ingredients so it’s not so complicated to fix a quick meal at home” (C-3).

#### 3.3.2. Spread Home Cooking to Other Family Members

Throughout the three time periods, participants from both groups said that, following the program, they cooked more also for family members who joined the effort to improve health through nutrition: “As the main cook at home, my husband really enjoys my cooking and thanks to them he also started to maintain a healthier diet” (C-2); “My wife was an advocate of ready-made and cheap food, …we finally managed to stop buying this food… and we rely quite a lot on homemade food” (C-3); “The new food I cooked during the program excites the whole family (pies, salads with different nuances, vegetarian meatballs)” (A-2); “I did enjoy cooking new foods. My family also enjoyed the new dishes that I cooked” (A-2).

However, two participants from the contemplation SOC reported on the challenge of spreading healthy cooking to the family: “Any time that a dish came out of the frizzier or fridge, I had three kids that dislike what I did and said: ‘we don’t eat this’, so I didn’t use the new tools… also when I tried to make a portion of the meal ahead of time it didn’t catch and wasn’t effective” (C-3); “The children had difficulty accepting the new dishes although they were happy to have cooked food more evenings a week” (C-2).

#### 3.3.3. Improve Nutrition Habits Throughout the Day

Through all three visits, participants from both groups reported improved nutritional habits throughout the day. Examples included eating healthier breakfasts and lunches and healthier snacking: “I stopped snacking in the evening… [I have] control over the amount of food I eat… I hope that control will become part of my lifestyle” (C-4); “I try to have a better breakfast and eat healthier snacks. Would have a bagel and cream cheese on most days before and do not eat that any longer” (A-4).

Specifically, participants reported improved nutrition at their workplace: “The added value for me was to change dietary habits, for example, bringing food for lunch when I work at school, something that did not happen before the program” (C-2); “… one of my big goals was to stop eating from the vending machine at school and bringing my own snacks and things like that, and that was something that I was able to accomplish” (C-3); “… prepare food for work… make time for food at the workplace. Stop everything, which is very difficult. Stop and say, ‘now I eat’.” (C-3); “… dinner salads have become more prevalent” (C-4).

Participants also described a more comprehensive nutritional change: “…. For many years, I walked around feeling hungry … today I eat ‘real meals’-that’s what I call them. Good, cooked meals. I feel like I’m eating nutritious things. I do everything by myself, cook a lot, eat nutritious food, feel full, satisfied” (A-3); “I got good ideas for satisfying myself without eating bread or cracker salt, seeds and yogurt and fruits, that was very helpful too… Those were the most important changes I made” (C-3).

#### 3.3.4. Decrease Consumption of Ultra-Processed Food

In both groups and throughout the three periods, participants say that, following the program, they consumed less ultra-processed food and cooked more from scratch: “I am trying to stay away from the processed food, to eat more food, which I do, trying to eat more nuts and healthier. For the most part, that happens” (A-3); “[Cooking] … without frying, eating mainly non-processed foods, using legumes, and especially home-made dishes based on vegetables, legumes. The smallest like the fruits I used to eat” (C-4). One participant shared that s/he “Learned to make things I would have bought (hummus, bread)” (C-2).

### 3.4. Address the Sustainability of Health Changes

#### 3.4.1. Achievements in Maintaining Long-Term Health Changes

Participants who entered in the active SOC mostly talked about their achievements up to one year from baseline. They seem confident that all they acquired in the program will have a long-term impact: “The cooking skills and ease will stay with me” (A-2); “In this program, I simply set goals and progressed, and things were kept. So, an amazing experience, very good” (A-3); “Definitely, the program has changed a lot in my life. I look at myself today after six months from the start of the program and three months since I finished the once-a-week talks and I see that I am still persistent in my whole diet plan. It has really been assimilated into me” (A3).

One participant also managed to change his self-perception, including his self-care: “The most significant moment in the program for me was the reminder that I am responsible for what happens to me and everything is in my hands. For some reason I forgot that over the past few years. Almost any goal I set for myself, I can achieve or at least make progress towards achieving it” (A-2).

#### 3.4.2. Challenges in Maintaining Long-Term Health Changes

Participants who entered in the contemplated SOC talked extensively throughout the year about the challenges in sustaining the new behaviors they acquired (i.e., home cooking), including their ability to maintain their achievements and satisfaction: “The main challenge was being consistent and committed to the changes I’m making” (C-2); “… My challenge for the future is not to slip into bad habits when I return to normal working routine to maintain what I have done throughout the study” (C-2). Participants also noted the difficulty in maintaining health outcomes such as their weight. Six months from baseline, participants emphasized the challenge for sustainability: “… I wish for the day that it will permeate me, be a part of me, that I will know when to stop when to say no to a cookie, dessert, or just to taste. Because that’s where I fall. In desserts, in carbohydrates” (C-3); “… I can’t tell you that it will stay forever, so if I feel that something is loosening, I try to strengthen it” (C-3).

#### 3.4.3. Facilitators for a Long-Term Change

Participants reported on key facilitators for sustainability: (1) Program facilitator “Having the chef coaching, I believe, helped out. She helps to keep me with my goals and help me be accountable. Never judging” (C-2); “I wanted to continue [with the coach] not in a once-a-week format. I’m sure that if she would accompany me even once a month, my day-to-day attention [to the subject] would have improved…” (C-3); “These talks gave me motivation, and it worked. As soon as the talks ended, the motivation was probably also lost…” (C-3); “I think the most helpful part was the motivation from the coach to stay on top of everything I was trying to do in terms of cooking and weight loss” (C-3); “… since the program end, I think the main difficulty is not having weekly motivating meeting with the coach” (C-3). (2) The need to report “What happened after the end of the program? I just fell apart. I returned to my previous weight… it seems funny, ridiculous… if they said… ‘You must report to the WhatsApp group’, no matter who, every week… just three sentences … It was a kind of eye watching me. If you want to change habits, you must know it’s not one habit… You have to give it more time than X meetings we had” (C-3); “I think a couple of check-ins maybe, and at the end of the program maid of 3 months plan and checked-in like once a month on it because some of my eating habits are changed” (C-3). (3) Being part of a framework “…I came to the insight that I must be part of a framework to see successes in the process. For example, consistency in drinking a lot of water during the day was achieved in this process” (C-2); “I understand the importance of being part of a framework that can help my family” (A-2).

Suggestions for future programs included spreading the meetings to a longer timeframe: “The idea of checking in with someone and then to keep a list of my questions. So, I didn’t like that it was 12 weeks. It was intense, and then it just stopped” (C-3); “I think I would stretch out so it’s the similar visits with the chef but spread out so that would be two weeks instead of every week because sometimes you’d have a conversation and then for example, you wouldn’t go to grocery shopping. Even between the next meetings, so you can kind of cheat…” (C-3); “The frequency of the meeting can decrease toward the end of the program, so it will last longer and will get used to continue on my own” (A-2).

## 4. Discussion

This study presents one-year perceptions of participants who enrolled in a home-cooking program at either a contemplation or action SOC, which has been shown to be the two SOCs with most engagement in cooking programs [20]. While all participants reported acquiring knowledge and skills, the contemplated participants were more focused on basic nutritional information and the use of recipes for cooking. They also asked for more practical, hands-on cooking workshops. All participants reported improved organization in the kitchen and confidence, which is an identified facilitator for adopting cooking behavior [20,21,22]. Participants also described having improved eating behaviors and nutrition, including adopting home-cooking habits and decreasing the consumption of UPF. However, while participants who entered the study in the action SOC were focused on their achievements, the contemplated participants reported challenges for sustainability and desire for extended support and reinforcements. Interestingly, participants in both groups reported nutritional achievements not directly related to increased home cooking such as decreased night snacking and making time for lunch.

In a previous report, we described goals and expectations from home-cooking intervention [39]. Participants who entered a home-cooking program in both the action and contemplation SOCs described interest in adopting sustainable home-cooking habits to achieve healthy eating and lifestyle goals [20]. This report is consistent with the progress of participants in the action and contemplation SOCs toward sustainable home-cooking habits. While the need for an onsite teaching kitchen for nutrition education is widely discussed [12], this report shows that expectation for health-related home-cooking education can be met with a low-budget telemedicine model. Before the program, only the contemplated participants expressed interest in improving self-regulatory skills [39]; however, this report shows that all participants enjoyed improved organizing and planning in the kitchen, which is also deemed important in other studies [20,22]. While contemplated participants reported acquiring specific tools, the action participants reported a broad success. This emphasizes the importance of including self-regulatory content [21] in addition to culinary training in CM programs [33] that might be specific to participants’ SOCs at baseline.

This home-cooking telemedicine intervention was based on health-coaching principles [45]. Consistent with the coaching literature, most of participants’ health behaviors maintained for up to one year [46]. However, while health coaching is a patient-centered intervention that addresses participant goals [47], this report emphasizes that there might be tools and topics that are important for participant success that may not be brought by participants. For example, while only the contemplated participants reported the expectation of improving self-regulatory skills at baseline, participants from both groups highlighted the importance of improving these skills in all three visits after the program. This emphasizes that addressing participant agenda is important but may not be sufficient. Further research is required on the benefit of tailoring home-cooking and coaching interventions to patient needs based on the science of change, such as the SOC theory. In addition, programs should account for the heterogeneity in cooking attitudes as facilitators for home cooking [22] and consider the person’s internal factors [24], including perceptions about cooking to promote sustainability. Potential modifications include the number of meetings and the interval between sessions, as well as the culinary and self-regulatory skills that enhance sustainability.

Participant perceptions are consistent with the literature that presents several themes that promote long-term behavior change, including maintenance motives, self-regulation [21], resources, and habit [20,48]. Participants from both the action and contemplation SOCs seem to have similar maintenance motives: the health benefits of home cooking and healthy nutrition. However, regarding self-regulation, while the effective strategy of contemplated participants was focused on specific tools, the action participants were more focused on joy and creativity. Considering resources, participants in both groups seemed to look for more physical resources (i.e., cooking workshops) and psychological resources (i.e., more meetings with the provider).

This study’s outcomes are also consistent with the POC theory [30,34]. Participants in the contemplation SOC described several experimental POCs. These includes recognizing vending machines as unhealthy stimuli (i.e., conscious raising), reduced worry from cooking mistakes (i.e., dramatic relief), identifying an increase in their confidence to cook (i.e., self-reevaluation), and recognizing the contribution of other members of the household and UPF consumption (i.e., environmental reevaluation) [30,34]. Thus, potential strategies in home-cooking programs for contemplated participants might include feedback about the current pattern of behavior and barriers to success; information about costs and harms of problem behaviors; imagining and visualizing benefits of change; mindfulness about strengths and weaknesses and emotions around the problem behavior; grieving losses and managing anger, fear, joy, and strength to promote positive change; clarifying one’s values for healthy cooking; creating new positive self-image; and considering the effect of one’s behavior on others in the household [20].

Participants in the action SOC described several behavioral POCs [30,34] such as trying to be more accountable and creative (i.e., self-liberation), using meal planning (i.e., counter conditioning), staying away from processed food (i.e., stimulus control), and excitement from the new food (i.e., reinforcement management). Thus, potential strategies in home-cooking programs for action participants might include the following: goal setting and plans to implement home cooking; practicing new decision-making strategies and commitment enhancement techniques; adopting new thinking and acting using healthy emotional regulation and coping such as acceptance and positive affirmations; instituting reminders and cues to make good decisions; removing triggers; building contingency contracts and covertly or overtly self-reward; and celebrating progress in healthy ways.

Strengths of this study include a binational cohort and a one-year follow-up. While the live remote delivery of the culinary coaching is an additional strength, further studies are needed to explore participant experiences following onsite CM programs. In addition, studies noted that the ability to make dietary changes can be affected by a caregiver [39], and this study only included participants who were the primary food providers. Limitations include a lack of participant socioeconomic diversity and the predominance of employed women who either married or live together with an above-the-average income. Gender disparities and social determinants of health largely affect people’s interest and concerns for food and nutrition [18,19,49]. This can be part of the reason for this CM’s success since known barriers to healthy home cooking included having less money and lower access for healthy food [22,24]. This study took place in a unique time, the COVID-19 pandemic, which included unique challenges and motivators to home cooking [50], which might have impacted the study participants. More studies are needed to explore whether further tailoring intervention according to SOCs will result in improved home cooking and health outcomes.

This study also has a small number of participants in the action SOC. Limitations of using SOC theory are that SOC is assessed by self-report, and change is not a linear process as individuals often recycle through stages before achieving long-term maintenance [51]. Another common limitation is that SOC theory does not account for social contexts such as socioeconomic status and income [22,24]. Prior systematic reviews suggest that determinants of home cooking are complicated and include cultural background and identities, household composition, socioeconomic status, health conditions, and aspirations [19]. These complexities demand a very personalized approach to improving the quality and quantity of home cooking and attention to both the processes and the outcomes of cooking programs. Practical aspects such as food access, storage, and money to purchase food can cause inequities between people with lower income and fewer opportunities. Therefore, further research is needed to determine how CM programs should consider wider psychological, environmental, and social factors related to home cooking.

## 5. Conclusions

The importance of reducing UPFs [52,53,54] and increasing home cooking [55,56] is becoming a nutritional priority [57], and culinary medicine programs that aim to improve home cooking are becoming popular [11]. These results provide one-year-long information about participant facilitators, barriers, and efficient tools for making home-cooking changes categorized to the participant stage of change at program entry. While more research in this area is needed, this information can help reform effective patient-centered culinary medicine programs.

## Figures and Tables

**Table 1:** Demographic of intervention group participants in the culinary coaching study.

	(n = 24)
Mean age, years (SD)	44.17 (12.92)
Female	17 (71%)
Male	7 (29%)
Marital Status	
Married	10 (42%)
Living together	4 (17%)
Never married	8 (33%)
Separated	1 (4%)
Divorced	1 (4%)
US Ethnic background	
American Indian	1 (8%)
Asian	2 (17%)
Black American African	0 (0%)
White	9 (75%)
Israel ethnic background	
Jewish	12 (100%)
Employment status	
Employed	21 (88%)
Retired	1 (4%)
Student	1 (4%)
Unemployed	1 (4%)
Yearly household income	
Below the average	2 (8%)
Around the average	6 (25%)
Above the average	16 (67%)
Highest level of education	
High school degree	0 (0%)
Bachelor’s degree or higher	23 (96%)
Other	1 (4%)

**Table 2:** Themes and sub-themes and examples of perspectives up to one year from baseline.

Themes	Sub-Themes	Visit 2 (After the Program)	Visit 3 (6-Month from Baseline)	Visit 4 (12 Months from Baseline)
Section 3.1 Acquire culinary and nutritional knowledge	Improve knowledge about healthy nutrition	“I learned to choose foods based on nutrition” (C-2).	“I finally learned how to prepare healthy food and check the ingredients I put in the pot. Not to put everything in the supermarket labeled ‘healthy’ or has a ‘healthy’ stigma… I am thirsty for more” (C-3).	“Probably more olive oil. More focus on vegetables and whole grains. Just a renewed focus on healthy eating” (A-4).
	Use new recipes	“I accomplished my goals…. with adding 1–2 new go-to recipes” (C-2).	“I think finding recipes, it came like a big thing for me … I found it on my own, go to recipes cook, things that I knew are good and I knew that I could cook easily and quickly… I have a little bit more verity…” (C-3).	“I also find that one of the things that has helped me the most is just looking up recipes on New York Times cooking and improvising” (A-4).
	Ask for hands-on cooking classes *	“It wasn’t necessarily cooking class or cooking coaching; the culinary coaching was not what I expected. I thought I was getting culinary techniques and classes” (C-2).	“I want health-oriented workshops, food that is good, and techniques on how to make it” (C-3).	--
Section 3.2 Improve culinary and self-regulatory skills	Improved confidence in the kitchen	“The success I’ve had, and the positive momentum will stay with me. The weekly goal setting and the resources I found as a result; and my confidence that I can cook healthy” (C-2).	“I think it helps with my confidence a lot, in terms of being able to cook healthier meals for myself and what to buy, like, where to find resources for recipes and things like that…” (C-3).	“The confidence that I have developed to know that I am able to cook healthy meals” (C-4).
	Expand cooking skills	“I started steaming (something I was not doing before) and grilling more” (C-2).	“I got a lot better at baking, and my knife skills got better. I also know better to making sauces I like” (A-3).	“When cooking, use onions, zucchini, pumpkin and other low-calorie vegetables instead of carbohydrates” (C-4).
	Organizing and planning	“Planning meals in advance… so that the ingredients are at home and the time needed for cooking is available. This means to plan already from the weekly shopping what I prepare that week” (C-2).	“I use a meal planning tool; I’m planning all my meals for the week. That’s one thing I never did before, and I frequently use now… I do meal prep in advance” (C-3).	“Incorporating prepared/frozen items into cooking at home that are consistent with the study (e.g., frozen cooked brown rice, vegetable medleys, etc.)” (C-4).
	Creativity and pleasure **	“I learned that I could make any changes I want to recipes to use all the food in my fridge, (i.e., substitute spinach for kale and chard)” (A-2).	“It mainly knows how to cook, reinventing yourself in the kitchen…” (A-3).	“…cooking skills will stay with me as will my confidence and newly found enjoyment of cooking. I cook as a hobby now” (A-4).
Section 3.3 Adopt home cooking and healthy nutrition	Adopting home-cooking habits	“Learned to make things I would have bought (hummus, bread)” (C-2).	“The whole idea around cooked food is what I learned… if it’s a real meal, it’s more satisfying, gives more of a feeling that we have eaten and there is no need to eat in between… I’ve been cooking more since participating in the research” (C-3).	“…I prepare most of my foods now where in the past I ordered out” (C-4).
	Spreading home cooking to other family members	“The new food I cooked during the program excites the whole family (pies, salads with different nuances, vegetarian meatballs)” (A-2).	“My wife was an advocate of ready-made and cheap food, … we finally managed to stop buying this food… and we rely quite a lot on homemade food” (C-3).	“Even though we [the family] now can get take-out, I’m much less interested in it. We will go back to eating out when we can, but that will be more for the social aspect, and I will aim to order more smartly” (C-4).
	Improve nutrition habits throughout the day	“The added value for me was to change dietary habits, for example, bringing food for lunch when I work at school, something that did not happen before the program” (C-2).	“… one of my big goals was to stop eating from the vending machine at school and bringing my own snacks and things like that, and that was something that I was able to accomplish” (C-3).	“I stopped snacking in the evening… [I have] control over the amount of food I eat… I hope that control will become part of my lifestyle” (C-4).
	Decrease consumption of ultra-processed food	“I learned to make things I would have bought (hummus, bread)” (C-2).	“I am trying to stay away from the processed food, to eat more food, which I do, trying to eat more nuts and healthier. For the most part, that happens” (A-3).	“[Cooking] … without frying, eating mainly non-processed foods, using legumes, and especially home-made dishes based on vegetables, legumes. The smallest like the fruits I used to eat” (C-4).
Section 3.4. Address the sustainability of health changes	Achievements in maintaining long-term health changes	“The cooking skills and ease will stay with me” (A-2).	“In this program, I simply set goals and progressed, and things were kept. So, an amazing experience, very good” (A-3).	“The routine of cooking at home is well established;” (C-4).
	Challenges in maintaining long-term health changes ***	“The main challenge was being consistent and committed to the changes I’m making” (C-2).	“… I can’t tell you that it will stay forever, so if I feel that something is loosening, I try to strengthen it” (C-3).	“Planning ahead and deciding not purchase certain foods like cookies and chips” (C-4).
	Facilitators for a long-term change	Visit 2: “Having the chef coaching, I believe, helped out. She helps to keep me with my goals and help me be accountable. Never judging” (C-2).	“These talks gave me motivation, and it worked. As soon as the talks ended, the motivation was probably also lost…” (C-3).	“Being in a nutritional/sports setting. Alone, I am not recruited” (C-4).

Themes and sub-themes of participants form both the contemplation and action stage of change. * Sub-theme of participants from only the contemplation group and one participant from action group/visit 2. ** Sub-theme of participants from the action group only. *** Sub-themes of participants form the contemplation group and one from action group through all visits.

## Data Availability

The datasets used and/or analyzed during the current study are available from the corresponding author on reasonable request.

## Supplementary Materials

The following supporting information can be downloaded at: https://www.mdpi.com/article/10.3390/nu17040704/s1, File S1: Qualitative Questionnaires.

## Abbreviations

The following abbreviations are used in this manuscript:

CM	Culinary Medicine
URICA	University of Rod Island Change Assessment Scale
SOC	Stage of Change
POC	Process of Change
UPF	Ultra-Processed Food

## References

[1] Dinu M., Pagliai G., Casini A., Sofi F. (2018). Mediterranean diet and multiple health outcomes: An umbrella review of meta-analyses of observational studies and randomized trials. Eur. J. Clin. Nutr..

[2] Guasch-Ferré M., Willett W.C. (2021). The Mediterranean diet and health: A comprehensive overview. J. Intern. Med..

[3] Micha R., Shulkin M.L., Peñalvo J.L., Khatibzadeh S., Singh G.M., Rao M., Fahimi S., Powles J., Mozaffarian D. (2017). Etiologic effects and optimal intakes of foods and nutrients for risk of cardiovascular diseases and diabetes: Systematic reviews and meta-analyses from the Nutrition and Chronic Diseases Expert Group (NutriCoDE). PLoS ONE.

[4] The World Health Organization, Healthy Diet. https://www.who.int/initiatives/behealthy/healthy-diet.

[5] The Academy of Nutrition and Dietetics, Cardiovascular Health/Heart Disease/Hypertension. https://www.eatright.org/health/health-conditions/cardiovascular-health-heart-disease-hypertension.

[6] Hayek S., Tessler R., Bord S., Endevelt R., Satran C., Livne I., Khatib M., Harel-Fisch Y., Baron-Epel O. (2019). Do Israeli health promoting schools contribute to students’ healthy eating and physical activity habits?. Health Promot. Int..

[7] Bisogni C.A., Jastran M., Seligson M., Thompson A. (2012). How people interpret healthy eating: Contributions of qualitative research. J. Nutr. Educ. Behav..

[8] Arlinghaus K.R., Johnston C.A. (2017). Advocating for Behavior Change with Education. Am. J. Lifestyle Med..

[9] Teixeira P.J., Silva M.N., Mata J., Palmeira A.L., Markland D. (2012). Motivation, self-determination, and long-term weight control. Int. J. Behav. Nutr. Phys. Act..

[10] Polak R., Tirosh A., Livingston B., Pober D., Eubanks J.E., Silver J.K., Minezaki K., Loten R., Phillips E.M. (2018). Preventing Type 2 Diabetes with Home Cooking: Current Evidence and Future Potential. Curr. Diab. Rep..

[11] Polak R., Phillips E.M., Nordgren J., La Puma J., La Barba J., Cucuzzella M., Graham R., Harlan T., Burg T., Eisenberg D. (2016). Health-related culinary education: A summary of representative emerging programs for health professionals and patients. Glob. Adv. Health Med..

[12] Eisenberg D.M., Burgess J.D. (2015). Nutrition Education in an Era of Global Obesity and Diabetes: Thinking Outside the Box. Acad. Med..

[13] Surgenor D., Hollywood L., Furey S., Lavelle F., McGowan L., Spence M., Raats M., McCloat A., Mooney E., Caraher M. (2017). The impact of video technology on learning: A cooking skills experiment. Appetite.

[14] Polak R., Finkelstein A., Paganoni S., Welch R., Silver J.K. (2019). Cooking Online with a Chef: Health Professionals’ Evaluation of a Live Culinary Coaching Module. Nutr. Metab. Insights.

[15] Polak R., Dill D., Abrahamson M.J., Pojednic R.M., Phillips E.M. (2014). Innovation in diabetes care: Improving consumption of healthy food through a “chef coaching” program: A case report. Glob. Adv. Health Med..

[16] Polak R., Pober D.M., Budd M.A., Silver J.K., Phillips E.M., Abrahamson M.J. (2017). Improving patients’ home cooking—A case series of participation in a remote culinary coaching program. Appl. Physiol. Nutr. Metab..

[17] Starke A.D., Dierkes J., Lied G., Kasangu G.A., Trattner C. (2025). Supporting healthier food choices through AI-tailored advice: A research agenda. PEC Innov..

[18] Taillie L.S. (2018). Who’s cooking? Trends in US home food preparation by gender, education, and race/ethnicity from 2003 to 2016. Nutr. J..

[19] Mills S., White M., Wrieden W., Brown H., Stead M., Adams J. (2017). Home food preparation practices, experiences and perceptions: A qualitative interview study with photo-elicitation. PLoS ONE.

[20] Garvin T.M., Chiappone A., Boyd L., Stern K., Panichelli J., Edwards Hall L.A., Yaroch A.L. (2019). Cooking Matters Mobile Application: A meal planning and preparation tool for low-income parents. Public Health Nutr..

[21] Di Noia J., Contento I.R., Prochaska J.O. (2008). Computer-mediated intervention tailored on transtheoretical model stages and processes of change increases fruit and vegetable consumption among urban African American adolescents. Am. J. Health Promot..

[22] Wolfson J.A., Bleich S.N., Smith K.C., Frattaroli S. (2015). What does cooking mean to you? perceptions of cooking and factors related to cooking behavior. Appetite.

[23] Mills S., White M., Brown H., Wrieden W., Kwasnicka D., Halligan J., Robalino S., Adams J. (2017). Health and social determinants and outcomes of home cooking: A systematic review of observational studies. Appetite.

[24] McGowan L., Caraher M., Raats M., Lavelle F., Hollywood L., McDowell D., Spence M., McCloat A., Mooney E., Dean M. (2017). Domestic cooking and food skills: A review. Crit. Rev. Food Sci. Nutr..

[25] Jones S.A., Walter J., Soliah L., Phifer J.T. (2014). Perceived motivators to home food preparation: Focus group findings. J. Acad. Nutr. Diet..

[26] Reicks M., Trofholz A.C., Stang J.S., Laska M.N. (2014). Impact of cooking and home food preparation interventions among adults: Outcomes and implications for future programs. J. Nutr. Educ. Behav..

[27] Reicks M., Kocher M., Reeder J. (2018). Impact of Cooking and Home Food Preparation Interventions Among Adults: A Systematic Review (2011–2016). J. Nutr. Educ. Behav..

[28] MacLean P.S., Wing R.R., Davidson T., Epstein L., Goodpaster B., Hall K.D., Levin B.E., Perri M.G., Rolls B.J., Rosenbaum M. (2015). NIH working group report: Innovative research to improve maintenance of weight loss. Obesity.

[29] Lavelle F., McGowan L., Spence M., Caraher M., Raats M.M., Hollywood L., McDowell D., McCloat A., Mooney E., Dean M. (2016). Barriers and facilitators to cooking from ‘scratch’using basic or raw ingredients: A qualitative interview study. Appetite.

[30] Plotnikoff R.C., Lippke S., Johnson S.T., Courneya K.S. (2010). Physical activity and stages of change: A longitudinal test in types 1 and 2 diabetes samples. Ann. Behav. Med..

[31] Spencer L., Wharton C., Moyle S., Adams T. (2007). The transtheoretical model as applied to dietary behaviour and outcomes. Nutr. Res. Rev..

[32] Vilamala-Orra M., Vaqué-Crusellas C., Foguet-Boreu Q., Guimerà Gallent M., Del Río Sáez R. (2021). Applying the Stages of Change Model in a Nutrition Education Programme for the Promotion of Fruit and Vegetable Consumption among People with Severe Mental Disorders (DIETMENT). Nutrients.

[33] Gordali M., Bazhan M., Ghaffari M., Omidvar N., Rashidkhani B. (2021). The effect of TTM-based nutrition education on decisional balance, self-efficacy and processes of change for fat intake. Health Educ..

[34] Prochaska J.O., DiClemente C.C. (1983). Stages and processes of self-change of smoking: Toward an integrative model of change. J. Consult. Clin. Psychol..

[35] Prochaska J.O., DiClemente C.C., Norcross J.C. (1992). In search of how people change. Appl. Addict. Behaviors. Am. Psychol..

[36] O’Brien B.C., Harris I.B., Beckman T.J., Reed D.A., Cook D.A. (2014). Standards for reporting qualitative research: A synthesis of recommendations. Acad. Med..

[37] McConnaughy E.N., Prochaska J.O., Velicer W.F. (1983). Stages of change in psychotherapy: Measurement and sample profiles. Psychother. Theory Res. Pract..

[38] Tambling R.B., Ketring S.A. (2014). The R-URICA: A confirmatory factor analysis and a revision to the URICA. Contemp. Fam. Ther..

[39] Polak R., Finkelstein A., Budd M.A., Gray B.E., Robinson H., Silver J.K., Faries M.D., Tirosh A. (2023). Expectations from a Home Cooking Program: Qualitative Analyses of Perceptions from Participants in “Action” and “Contemplation” Stages of Change, before Entering a Bi-Center Randomized Controlled Trial. Nutrients.

[40] Braun V., Clarke V. (2019). Reflecting on reflexive thematic analysis. Qual. Res. Sport Exerc. Health.

[41] Braun V., Clarke V. (2006). Using thematic analysis in psychology. Qual. Res. Psychol..

[42] Braun V., Clarke V., Hayfield N. (2022). ‘A starting point for your journey, not a map’: Nikki Hayfield in conversation with Virginia Braun and Victoria Clarke about thematic analysis. Qual. Res. Psychol..

[43] Polit D.F., Beck C.T. (2010). Generalization in quantitative and qualitative research: Myths and strategies. Int. J. Nurs. Stud..

[44] Tracy S.J. (2010). Qualitative quality: Eight “big-tent” criteria for excellent qualitative research. Qual. Inq..

[45] NBME Content Outline. https://www.nbme.org/sites/default/files/2022-05/NBHWC_Content_Outline.pdf.

[46] Appel L.J., Clark J.M., Yeh H.C., Wang N.Y., Coughlin J.W., Daumit G., Miller I.I.I.E.R., Dalcin A., Jerome G.J., Geller S. (2011). Comparative effectiveness of weight-loss interventions in clinical practice. N. Engl. J. Med..

[47] Smith L.L., Lake N.H., Simmons L.A., Perlman A., Wroth S., Wolever R.Q. (2013). Integrative Health Coach Training: A Model for Shifting the Paradigm Toward Patient-centricity and Meeting New National Prevention Goals. Glob. Adv. Health Med..

[48] Kwasnicka D., Dombrowski S.U., White M., Sniehotta F. (2016). Theoretical explanations for maintenance of behaviour change: A systematic review of behaviour theories. Health Psychol. Rev..

[49] Stratten M., McGee-Brown J., Brinston T., Phipps S., Purry A. (2023). Engaging Black Men and Fathers in Supplemental Nutrition Assistance Program-Education (SNAP-Ed). J. Nutr. Educ. Behav..

[50] González-Monroy C., Gómez-Gómez I., Olarte-Sánchez C.M., Motrico E. (2021). Eating behaviour changes during the COVID-19 pandemic: A systematic review of longitudinal studies. Int. J. Environ. Res. Public Health.

[51] Norcross J.C., Krebs P.M., Prochaska J.O. (2011). Stages of Change. J. Clin. Psychol..

[52] Marfella R., Prattichizzo F., Sardu C., Fulgenzi G., Graciotti L., Spadoni T., D’Onofrio N., Scisciola L., La Grotta R., Frigé C. (2024). Microplastics and Nanoplastics in Atheromas and Cardiovascular Events. N. Engl. J. Med..

[53] Tobias D.K., Hall K.D. (2021). Eliminate or reformulate ultra-processed foods? Biological mechanisms matter. Cell Metab..

[54] Lane M.M., Gamage E., Du S., Ashtree D.N., McGuinness A.J., Gauci S., Baker P., Lawrence M., Rebholz C.M., Srour B. (2024). Ultra-processed food exposure and adverse health outcomes: Umbrella review of epidemiological meta-analyses. BMJ.

[55] Willett W., Rockström J., Loken B., Springmann M., Lang T., Vermeulen S., Garnett T., Tilman D., DeClerck F., Wood A. (2019). Food in the Anthropocene: The EAT–Lancet Commission on healthy diets from sustainable food systems. Lancet.

[56] Tan J., Atamanchuk L., Rao T., Sato K., Crowley J., Ball L. (2022). Exploring culinary medicine as a promising method of nutritional education in medical school: A scoping review. BMC Med. Educ..

[57] Lichtenstein A.H., Appel L.J., Vadiveloo M., Hu F.B., Kris-Etherton P.M., Rebholz C.M., Sacks F.M., Thorndike A.N., Van Horn L., Wylie-Rosett J. (2021). 2021 dietary guidance to improve cardiovascular health: A scientific statement from the American Heart Association. Circulation.

