# Supplementary material for: Personalized Culinary Medicine: Qualitative Analyses of Perceptions from Participants in Action and Contemplation Stages of Change Through a One-Year Bi-Center Randomized Controlled Trial
Source: Nutrients. 2025 Feb 16;17(4):704. doi: 10.3390/nu17040704 (PMC11858461; doi:10.3390/nu17040704)
Supplement: Supplementary file 1 [file nutrients-17-00704-s001.zip › nutrients-3460149-supplementary.pdf]

Supplementary File S1 - Qualitative Questionnaires

Visit 2- At the end of the CHEF Coaching program

1. How would you describe your experience during the CHEF Coaching program in general? What were your main challenges during the program? Did the program meet your expectations? Please provide examples that will illustrate your feelings and thoughts.
2. What are the most efficient CHEF Coaching tools and / or skills that you gained during the program? Please provide examples that will illustrate how these tools were beneficial for you.
3. What was unnecessary/not useful for you in the program? What could you change in the program and/ or add to the program? Please provide an example that will illustrate your feelings and thoughts.
4. Did you make use in the CHEF Coaching education resources and / or other videos? What were the advantages and disadvantages of these tools for you?
5. What changes (if any) do you recognize in yourself in relation to cooking as a result of the CHEF Coaching program? Do you recognize in yourself any other changes as a result of the program that are not directly related to program content? Please provide examples.
6. Did you enjoy the new food that you cooked during the program? What were the responses (if any) from people who ate the food that you cooked (family, friends, etc.)? Please provide examples that will illustrate your feelings and thoughts.
7. Can you recall a meaningful learning moment during the program? Please explain why it was meaningful and what did you take from it for the future.
8. Looking back at your nutritional goals before entering the program, which of them did you accomplish and which of them remain as a challenge for the future?
9. Looking to the future - What will stay with you from the program the most?

Visit 3 - Six months after the beginning of the program
Main leading questions

1. Looking back- how would you describe your experience in the CHEF Coaching program in general? What was the significant achievement for you? What was your main difficulty? Please provide an example that will illustrate your feelings and thoughts.
2. What are the most efficient culinary tools/skills that were especially successful / effective for you during the last 3 months since the CHEF Coaching program has ended? Please provide examples that will illustrate how these tools were beneficial for you.
3. Did you use any of the CHEF Coaching education resources and videos in the last 3 months since the CHEF Coaching program has ended? Please provide examples that will illustrate advantages and disadvantages of these tools.
4. Are there any new resources (i.e., recipes, videos) or new culinary tools that you started using since the end of the program? What are the tools that you learned during the program that helped you find the new resources or acquire the new skills?
5. What tools learned in the CHEF Coaching program were unnecessary and / or ineffective for you in the last 3 months?
6. Considering your cooking experience in the last 3 months since the program ended, what would you change and / or add to the program? Please give examples to illustrate your opinion.
7. Looking back at your nutritional goals before entering the CHEF Coaching program, which ones have you accomplished, and which ones remain as a challenge for the future?
8. Looking to the future - what will stay with you from the CHEF Coaching program the most?

Visit 4 – Twelve months after the beginning of the program

1. Please describe the culinary techniques and/or tool that were most effective for you during the last year since you have started the program.
2. Please describe your most important nutritional change since the beginning of the program. Have you adopted any other lifestyle changes in addition to nutritional changes? If yes, please explain these additional changes.
3. Looking back at your goals before entering the program, which of them did you accomplish?
4. Looking to the future - What will stay with you from the program the most?
